# The multitude of molecular analyses in cancer: the opening of Pandora’s box

**DOI:** 10.1186/s13059-014-0447-6

**Published:** 2014-09-03

**Authors:** Hege G Russnes, Per E Lønning, Anne-Lise Børresen-Dale, Ole C Lingjærde

**Affiliations:** Department of Genetics, Institute for Cancer Research, Oslo University Hospital, Postbox 4950, Nydalen 0424 Oslo, Norway; Department of Pathology, Oslo University Hospital, Postbox 4950, Nydalen 0424 Oslo, Norway; K.G. Jebsen Center for Breast Cancer Research, University of Oslo, Oslo, Norway; Section of Oncology, Department of Clinical Science, University of Bergen, Bergen, Norway; Department of Oncology, Haukeland University Hospital, Jonas Lies vei 26, 5021 Bergen, Norway; Biomedical Informatics division, Department of Computer Science, University of Oslo, Oslo, Norway

## Abstract

The availability of large amounts of molecular data of unprecedented depth and width has instigated new paths of interdisciplinary activity in cancer research. Translation of such information to allow its optimal use in cancer therapy will require molecular biologists to embrace statistical and computational concepts and models.

Progress in science has been and should be driven by our innate curiosity. This is the human quality that led Pandora to open the forbidden box, and like her, we do not know the nature or consequences of the output resulting from our actions. Throughout history, ground-breaking scientific achievements have been closely linked to advances in technology. The microscope and the telescope are examples of inventions that profoundly increased the amount of observable features that further led to paradigmatic shifts in our understanding of life and the Universe. In cell biology, the microscope revealed details of different types of tissue and their cellular composition; it revealed cells, their structures and their ability to divide, develop and die. Further, the molecular compositions of individual cell types were revealed gradually by generations of scientists. For each level of insight gained, new mathematical and statistical descriptive and analytical tools were needed (Figure 1a). The integration of knowledge of ever-increasing depth and width in order to develop useful therapies that can prevent and cure diseases such as cancer will continue to require the joint effort of scientists in biology, medicine, statistics, mathematics and computation.

Here, we discuss some major challenges that lie ahead of us and why we believe that a deeper integration of biology and medicine with mathematics and statistics is required to gain the most from the diverse and extensive body of data now being generated. We also argue that to take full advantage of current technological opportunities, we must explore biomarkers using clinical studies that are optimally designed for this purpose. The need for a tight interdisciplinary collaboration has never been stronger.

## Decomposing tumors into cell types and molecular alterations

Neoplastic transformation can start in nearly every cell type in the human body. It is recognizable as cells that have the ability to divide uncontrollably and to escape aging mechanisms and naturally occurring cell death, resulting in the growth of a tumor. Tumors have different features, depending on the organ of origin and the level of differentiation of the tumor cells. At certain points in development, a tumor will be influencing its microenvironment, ensuring, among other things, vascularization and cooperation with the immune system. A tumor can progress further, evolving into malignant disease, by invading the surrounding tissue, disseminating into the bloodstream or lymphatic channels, and establishing metastases in other parts of the body, often with fatal consequences for the affected individual. The facets of such transformations are linked to distinct biological processes, but these differ according to cell type (that is, the cell of origin), the local microenvironment, host factors such as an individual’s genetic background and age, and exogenous and endogenous environmental influences [1].

The diversity of cancer, in both biological and clinical terms, is well acknowledged and has been extensively studied. Today, with increasingly sophisticated technologies at our disposal, highly detailed molecular features of individual tumors can be described. Such features are often referred to as being layered, occurring at a genomic (DNA) level, a transcriptomic (mRNA) level and a functional (protein) level. The proteins are the key functional elements of cells, resulting from transcription of a gene into mRNA, which is further translated into protein. This simplistic way of describing the relationship between the layers has gradually changed during the past decades of functional and molecular insight. Protein synthesis is no longer perceived as a linear process, but as an intricate network of a multitude of operational molecules. Astonishing progress has been made in the discovery of molecules that are able to influence transcription and translation, such as DNA-modifying enzymes and non-translated RNAs, and of mechanisms that are able to control the processing, localization and activation of proteins [2,3].

A picture is emerging of individual cells within a tumor that can differ at the genomic, epigenomic and transcriptional levels, as well as at the functional level [4,5]. Mutations and epigenetic alterations create the required phenotypic diversity that, under the influence of shifting selective pressures imposed by the environment, determines the subclonal expansion and selection of specific cells. The development of solid tumors thus follows the same basic principles as Darwinian evolution. Most single nucleotide polymorphism (SNP) variants that arise in human evolution are neutral in respect to survival advantage; over a period of time, these variants are typically fixed in or die out from the genome according to chance. Other variants provide a survival advantage [6] and will, over time, dominate the cell population, leading to distinct haploid signatures. Cancer may involve hundreds or thousands of mutations, with each mutation potentially contributing to tumor fitness. Most of these mutations are assumed to be passengers, but a limited number have driver capability, sometimes only in a subpopulation of cells [7-9].

There is an intricate interplay between subpopulations of tumor cells and among tumor and normal cells in the microenvironment, and tumor topology is likely to play a role in this context. Our knowledge of molecular mechanisms in cancer development and progression are mainly derived from model systems such as *in vitro* cell cultures and animal models, as well as from descriptive molecular analyses of tissue samples. Model systems have been crucial for understanding molecular interactions and their implications in cancer, but they cannot fully mimic tumor conditions *in vivo*. Tissue samples, on the other hand, contain both a microenvironment and subpopulations of cancer cells, but they represent only a snapshot in an individual tumor’s life history. Until recently, cancer studies mainly considered only one or a few molecular levels at a time. Altered protein expression can have several causes [10]; it can be due to copy-number gain, a translocation event that combines the gene with an active promoter, alteration of factors that modify DNA or influence the transcription machinery, or modifications of mRNA or the protein itself. Revealing the various downstream effects of such alterations is potentially useful for tumor classification and for prediction of treatment response and prognosis [11].

The pathways that affect or are affected by tumor development need to be identified, but there may be little hope of intervening unless we know which molecular factors control the pathways in each patient. In addition, a key to a more fundamental understanding of the biological dynamics will be to consider tumors from a systems biology perspective. Systems biology seeks to understand a tumor as an interplay between various processes and external stimuli, the ultimate goal being to predict the effect of a perturbation of any part of the system [12]. Detailed studies of the regulatory networks and molecular interactions that take place in different types of cells under various conditions will be crucial for understanding the biological and clinical behavior of normal and malignant cells. This will require analyses of both large-scale omics data and deeply characterized data sets derived from functional studies, such as those developed in the LINCS project [13]. The importance of functional studies as a foundation for molecular diagnostic tools has been illustrated by a recent work in which a histone demethylase, JARID1b, was found to have an oncogenic function in breast cell lines that undergo luminal differentiation [14]. The detailed multilevel alterations induced by JARID1b were analyzed in a pathway-specific manner to develop a diagnostic test. The index thus designed was applied to a breast cancer dataset that included both DNA copy number and mRNA expression data, showing that inferred JARID1b activity was prognostic for estrogen-receptor-positive disease. A cell-type-specific functional understanding of molecular alterations will be increasingly important to improve the success of molecular assays in clinical decision-making.

## Bringing all the information together

**Figure 1:**
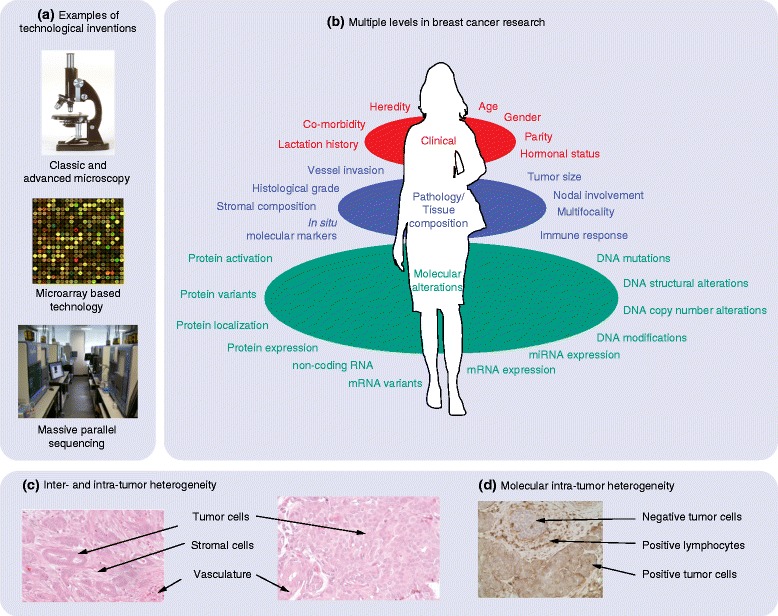
**Multiple levels of data integration. (a)** Major inventions resulting in extreme numbers of novel observations in cancer biology: the microscope, microarray analyses and massive parallel sequencing technology. **(b)** The gap between current breast cancer treatment and available molecular data. Most treatment guidelines are based on studies that analyzed a small number of measurements reflecting clinical information (red) and histopathological features (blue). By contrast, an almost unlimited number of measurements can be obtained in multilevel molecular analyses using, for example, microarray-based technologies or massive parallel sequencing technologies where millions of data points can be observed simultaneously (green). miRNA, microRNA. **(c)** Inter-tumor heterogeneity is illustrated by these microscopic images of two tumors, one having tumor cells with luminal differentiation and abundant stromal tissue (left) and the other having less differentiated tumor cells and scarcely any stromal tissue (right). **(d)** The cellular composition of tumors shows great variation, as do the tumors themselves. Immunohistochemistry with an antibody against the membrane protein CD44 shows both positive and negative stained tumor cells. In addition, lymphocytes in the stromal environment are positive for this antibody.

Standard breast cancer care is primarily based on former research employing clinical features, histopathology measurements and analyses of a handful of molecular interactions. Current technology allows advanced molecular characterizations of tumor samples at multiple layers and down to single cell resolution, thus dramatically increasing the number of measurements that can be obtained from clinical tumor samples (Figure 1b). The wealth of data from such massive parallel analyses represents a serious computational and interpretational challenge, but also new inferential opportunities.

In many ways, these developments in molecular biology reflect the progress in other natural sciences, including chemistry, physics and astronomy. For example, as distant astronomical objects were observed at an increasing number of wavelengths and at increasing resolution, more detailed models were gradually formulated of processes that had been visible even to Galileo, albeit at a much cruder scale. In much the same way, the various omics data and other molecular data now available will provide new perspectives on known processes in a tumor and its environment, allowing more detailed pictures to be drawn. The complexity of the biological system does not increase, only our ability to observe it and to build realistic models and make useful predictions.

Facing these challenges sometimes requires the rethinking or extension of well-established concepts and procedures. For example, when classical statistical hypothesis testing is applied to thousands of cases simultaneously, inaccurate specification of the null model can result in severe over- or under-reporting of significant cases. The very fact that many tests are performed in parallel, however, allows the null model to be empirically determined and thus corrected [15]. Another example concerns the determination of the significance threshold (the threshold used to decide whether a *P*-value is small enough to reject the null hypothesis) when many hypothesis tests are being performed and the benefit of detecting many real effects can justify a small proportion of false positives, a problem for which the false discovery rate (FDR) was invented [16].

Gaining the most from the rapidly growing flow of information is an intellectual enterprise as much as it is a technical one. Even the most skilled molecular biologist can no longer see the full consequences for existing hypotheses of a new piece of information, unless practical mechanisms are in place to assimilate and eventually integrate the new information with the wealth of existing knowledge. This will require extensive data sharing, more sophisticated statistical and bioinformatical tools for integrative analyses, mechanisms that promote shared analyses, and increasing computing power (see [17] and references therein). In addition, we need novel strategies for sharing hypotheses and models - an equivalent of the physicists’ standard model, which represents a common reference that novel results can be confronted with and challenge.

To achieve this goal, deeper integration between biology, mathematics and statistics should be sought by developing practical and sufficiently general frameworks for the formulation and testing of complex biological hypotheses. The benefits of having such frameworks are likely to grow rapidly as the complexity of observations and models increases. In addition, it will be important to be able to share analyses publicly in a standardized fashion; this is a serious challenge when the analysis is based on multiple tools running on different computing platforms, and both the tools and the platforms are subject to regular updates and dependencies on external data sources. Low-dimensional representations of biological systems, that is, representations that can be mathematically described using a small number of variables (5 to 10 or fewer), are also likely to play an important role in the future; if nothing else, they appeal to our intuition and ability to think conceptually. With more data and more molecular levels available, however, the low-dimensional representation (projection) can be judiciously selected to reflect the most relevant properties of the processes governing the behavior of the system under study. The rapid growth in computing power, novel statistical methodologies and computational tools that can handle datasets of increasing size and complexity gives further cause for optimism. As Galileo needed mathematics to describe and interpret his observations, today we need the theory and tools of mathematics and statistics to develop our understanding of life.

*[The universe] cannot be read until we have learnt the language and become familiar with the characters in which it is written. It is written in mathematical language, and the letters are triangles, circles and other geometrical figures, without which means it is humanly impossible to comprehend a single word.* Galileo Galilei

The question remains as to how to find the best projection in an ocean of irrelevant features that do nothing but increase the dimensionality of the problem. While most of statistics concerns some form of separation of signal from noise, the problems encountered when thousands of variables are involved, commonly called the curse of dimensionality, make the analysis of such data intractable with classical statistical techniques. From a geometric point of view, the main problem is that high-dimensional spaces are very large and the concept of localness, which is fundamental to a wide range of statistical methods, breaks down when they are analyzed.

The key to escaping the curse of dimensionality is, first, the realization that the actual number of parameters in a model, or its degrees of freedom, can be controlled by imposing constraints on those parameters. Thus, we may include thousands of variables in a regression model and obtain a sensible estimate, as long as we impose appropriate restrictions on the coefficients to be estimated. Second, under suitable conditions, such constraints have been shown to improve the estimate in a precise statistical sense [18]. Third, we have the practical means today to impose such constraints in a biologically sensible way, by defining precise assumptions (priors) on the model’s behavior that are based on available knowledge and observations. Such priors can, for example, be used to incorporate spatial information, such as where in the cell protein-protein interactions occur, or to build into a model knowledge of specific molecular interactions or functions. Knowledge that is used to define priors can come from other molecular levels, other patient materials, other cancer types, or normal specimens [19].

## From integrative models to cancer diagnostics and treatment

Yet to us all - scientists, physicians in charge of patient care and potential cancer patients - the ultimate question remains: how can the wealth of knowledge that is available be translated to improved patient treatment? For decades this question was considered to be an academic exercise of interest to scientists alone, but today, translation research is recognized as mandatory for the identification of mechanisms that are responsible for therapy resistance. The past decade of research has, for example, refined breast cancer classification to include data on gene expression and copy number alterations, and revealed the prognostic impact of molecular-based classification [20,21]. These examples, however, relate to prognostication: an overall outcome that is influenced by tumor biology and therapy effects in a way that generally does not allow dissection of the mechanism of sensitivity toward the therapeutic agents employed [22]. In breast cancer, emerging evidence suggests that tumors belonging to the ‘basal-like’ class have a particular sensitivity for platinum-containing compounds and in particular PARP inhibitors. This sensitivity relates to defects in a particular functional pathway (homologous repair) that characterizes these tumors in individuals who carry a *BRCA1* germline mutation; these defects may also affect other tumors within the ‘basal-like’ category [23].

Experimental evidence should be interpreted with caution; for example, the tumor suppressor gene *TP53* has been intensively studied for more than three decades, but new evidence relating to its role in cancer is continuously emerging. The importance of different mechanisms of drug action to the *in vivo* chemosensitivity of pathways that are affected by the *TP53* mutation remains unclear (see references in [24,25]). This illustrates the need for translational studies properly designed to address these questions. Although the endpoint from a therapeutic perspective is overall survival, endpoints like relapse-free as well as overall survival need to be addressed fully in the context of large, randomized phase III trials.

Over the past decade, studies carried out in neoadjuvant or presurgical therapy settings have employed parameters such as a drop in the cancer antigen KI67 during the first weeks on endocrine therapy. While parameters like having a pathological complete response or primary progression can still be used as surrogate endpoints for long-term outcome (see discussion in [26]), recent results [27] indicate that correlations between tumor shrinkage and long-term outcome are not working well in all clinical settings as exemplified by tumors in patients carrying a germline *BRCA1* mutation. There is a need for a systems biology approach to identify redundant pathways [28] and, in particular, to determine how such mechanisms may work differently in different tumor forms. These approaches must also consider the potential impact of the microenvironment on sensitivity to treatment. When administered to breast cancer patients on endocrine treatment or to patients harboring activating mutations in the phosphoinositide 3-kinase (PI3K) pathway, the mammalian target of rapamycin (mTOR) inhibitor everolimus improved outcome [29], but this drug was ineffective among patients whose tumors harbored mutations in redundant pathways [30]. Agents that target activating mutations, including the *BRAF* oncogene, have been shown to be highly effective in malignant melanomas; by contrast, these same agents work poorly in metastatic colorectal cancer because of a compensatory increase of epithelial growth factor receptor (EGF-R) activity [31]. Observations such as these should not provoke pessimism; they merely underline the need to implement the proper models and parameters in clinical studies.

As for improving outcome, studies in which tumor tissue specimens are collected before and during therapy should continue. Novel techniques, including massive parallel sequencing and different types of omics technologies, allow the study of tumor biomarkers in a way we could only dream of a decade ago, and make it possible to correlate these biomarkers to tumor regression in response to therapy. In parallel, samples must be collected during large phase III trials so that, in due time, we can develop well-annotated tumor banks that, when combined with clinical information, will be able to confirm the impact of molecular-based diagnostic tests on long-term outcome. Perhaps even more important is the collection of repeated samples from tumor tissue and circulating DNA during therapy to monitor clonal changes [32].

Studies on predictive biomarkers have traditionally measured alterations at initial diagnosis, prior to surgery and compared the presence of genetic mutations or other disturbances to clinical outcome defined by tumor shrinkage. Implementation of massive parallel sequencing, however, allows the estimation of biomarkers within a clonal setting, offering a unique possibility of evaluating changes in biomarkers during the course of therapy. For instance, if a certain gene mutation is detected among 80% of all cells at the initiation of therapy but disappears after three months of chemotherapy (independent of any tumor shrinkage), this marker is associated with cells that are therapy sensitive. Conversely, a biomarker identified in 10% of tumor cells prior to therapy but among 80% after therapy may be considered a marker of drug-resistant cells surviving therapy. While the issue of tumor heterogeneity should be taken into account, modern techniques of sampling allow several samples to be collected in parallel in a non-traumatic setting.

Finally, we should not forget the pathologists and the ability of ‘the old dogs to perform their old tricks’. Repeated histologic examinations, using techniques such as fluorescence *in-situ* hybridization (FISH), are required not only in the interest of confirming gene amplifications and assessing intra-tumor heterogeneity; for example, the beta-galactosidase assay could be applied to clinical samples to assess the potential importance of senescence (as outlined in animal models) to chemotherapy efficacy in human tumors [33].

## The spirit of hope

Understanding the biological mechanisms behind cancer requires the ability to identify biological processes in individual tumors and within the different cell types (Figure 1c,d), as well as to integrate a multitude of observations made at several molecular levels. It is an overwhelming task, but one that needs to be pursued along with the development of novel ways of combining and integrating scientific evidence across several molecular levels, study cohorts of various designs (adjuvant and neoadjuvant), many research groups and different diseases. Here, the novel developments in large-scale statistical inference and the empirical Bayes approach, which unifies aspects of the frequentist and Bayesian philosophies (Box 1), are likely to play a major role in the years to come.

Pandora opened her box impelled by her curiosity. What was left in Pandora’s box was the Spirit of Hope. In our context, this is the development of novel computational approaches, statistics and models combined with the ongoing pursuit of characterizing cancers of all different types and stages. The field would be well served by a concerted world-wide effort to make molecular profiles, associated clinical or biological data and analytics publicly available in standardized fashion that facilitates the development of analytics. Closer ties should be forged between biology, mathematics and statistics, thus moving away from the concept of applying mathematical and statistical tools to solve specialized tasks and towards a common interdisciplinary framework for expressing and testing biological hypotheses and models.

## Box 1: Frequentist versus Bayesian philosophies

The frequentist approach to statistics considers the probability of an event to be the relative frequency of that event in a large number of trials. According to this view, a statistical hypothesis is fixed and cannot be assigned a probability, while the data used to test it are considered to be random. The Bayesian approach to statistics views probabilities as quantities reflecting states of knowledge or belief, and probabilities can be assigned to statistical hypotheses. To determine the credibility of a null hypothesis, a frequentist would calculate the probability of the observed data given the hypothesis, whereas a Bayesian would calculate the probability of the hypothesis given the data.

Empirical Bayes combines elements of the Bayesian and frequentist points of view by allowing the priors used in Bayes analysis to be estimated from the data. Conceived of more than 60 years ago, it is only now, with the current generation of data sets involving a huge number of parallel experiments, that the full force of empirical Bayes is brought to bear. By offering the opportunity to build realistic, data-based biological assumptions into our statistical models, empirical Bayes will be a valuable tool for developing the next generation of integrative analysis methods.

## Abbreviations

EGF-R: Epithelial growth factor receptor
FDR: False discovery rate
FISH: Fluorescence *in-situ* hybridization
SNP: Single nucleotide polymorphism

## References

[1] Hanahan D, Weinberg RA (2011). Hallmarks of cancer: the next generation. Cell.

[2] Baylin SB, Jones PA (2011). A decade of exploring the cancer epigenome - biological and translational implications. Nat Rev Cancer.

[3] Morris KV, Mattick JS (2014). The rise of regulatory RNA. Nat Rev Genet.

[4] Marusyk A, Almendro V, Polyak K (2012). Intra-tumour heterogeneity: a looking glass for cancer?. Nat Rev Cancer.

[5] Russnes HG, Navin N, Hicks J, Borresen-Dale AL (2011). Insight into the heterogeneity of breast cancer through next-generation sequencing. J Clin Invest.

[6] Bamshad M, Wooding SP (2003). Signatures of natural selection in the human genome. Nat Rev Genet.

[7] Greenman C, Stephens P, Smith R, Dalgliesh GL, Hunter C, Bignell G, Davies H, Teague J, Butler A, Stevens C, Edkins S, O’Meara S, Vastrik I, Schmidt EE, Avis T, Barthorpe S, Bhamra G, Buck G, Choudhury B, Clements J, Cole J, Dicks E, Forbes S, Gray K, Halliday K, Harrison R, Hills K, Hinton J, Jenkinson A, Jones D (2007). Patterns of somatic mutation in human cancer genomes. Nature.

[8] Sjöblom T, Jones S, Wood LD, Parsons DW, Lin J, Barber TD, Mandelker D, Leary RJ, Ptak J, Silliman N, Szabo S, Buckhaults P, Farrell C, Meeh P, Markowitz SD, Willis J, Dawson D, Willson JKV, Gazdar AF, Hartigan J, Wu L, Liu C, Parmigiani G, Park BH, Bachman KE, Papadopoulos N, Vogelstein B, Kinzler KW, Velculescu VE (2006). The consensus coding sequences of human breast and colorectal cancers. Science.

[9] Nik-Zainal S, Alexandrov LB, Wedge DC, Loo PV, Greenman CD, Raine K, Jones D, Hinton J, Marshall J, Stebbings LA, Menzies A, Martin S, Leung K, Chen L, Leroy C, Ramakrishna M, Rance R, Lau KW, Mudie LJ, Varela I, McBride DJ, Bignell GR, Cooke SL, Shlien A, Gamble J, Whitmore I, Maddison M, Tarpey PS, Davies HR, Papaemmanuil E (2012). Mutational processes molding the genomes of 21 breast cancers. Cell.

[10] Myhre S, Lingjærde OC, Hennessy BT, Aure MR, Carey MS, Alsner J, Tramm T, Overgaard J, Mills GB, Børresen-Dale AL, Sørlie T (2013). Influence of DNA copy number and mRNA levels on the expression of breast cancer related proteins. Mol Oncol.

[11] Brodtkorb M, Lingjærde OC, Huse K, Trøen G, Hystad M, Hilden VI, Myklebust JH, Leich E, Rosenwald A, Delabie J, Holte H, Smeland EB (2013). Whole-genome integrative analysis reveals expression signatures predicting transformation in follicular lymphoma. Blood.

[12] Ram PT, Mendelsohn J, Mills GB (2012). Bioinformatics and systems biology. Mol Oncol.

[13] ᅟ (ᅟ). NIH LINCS program. ᅟ.

[14] Yamamoto S, Wu Z, Russnes HG, Takagi S, Peluffo G, Vaske C, Zhao X, Moen Vollan HK, Maruyama R, Ekram MB, Sun H, Kim JH, Carver K, Zucca M, Feng J, Almendro V, Bessarabova M, Rueda OM, Nikolsky Y, Caldas C, Liu XS, Polyak K (2014). JARID1B is a luminal lineage-driving oncogene in breast cancer. Cancer Cell.

[15] Efron B (2004). Large-scale simultaneous hypothesis testing: the choice of a null hypothesis. J Am Stat Assoc.

[16] Benjamini Y, Hochberg Y (2005). Controlling the false discovery rate: a practical and powerful approach to multiple testing. J R Stat Soc Series B Stat Methodol.

[17] Kristensen VN, Lingjærde OC, Russnes HG, Vollan HKM, Frigessi A, Børresen-Dale AL (2014). Principles and methods of integrative genomic analyses in cancer. Nat Rev Cancer.

[18] Stein C (1955). Inadmissibility of the Usual Estimator for the Mean of a Multivariate Normal Distribution. Proceedings of the Third Berkeley Symposium on Mathematical Statistics and Probability.

[19] Stingo FC, Vannucci M, Do K-A, Qin ZS, Vannucci M (2013). Bayesian Models for Integrative Genomics. Advances in Statistical Bioinformatics.

[20] Sørlie T, Perou CM, Tibshirani R, Aas T, Geisler S, Johnsen H, Hastie T, Eisen MB, van de Rijn M, Jeffrey SS, Thorsen T, Quist H, Matese JC, Brown PO, Botstein D, Lønning PE, Børresen-Dale AL (2001). Gene expression patterns of breast carcinomas distinguish tumor subclasses with clinical implications. Proc Natl Acad Sci U S A.

[21] Curtis C, Shah SP, Chin SF, Turashvili G, Rueda OM, Dunning MJ, Speed D, Lynch AG, Samarajiwa S, Yuan Y, Gräf S, Ha G, Haffari G, Bashashati A, Russel R, McKinney S, Group M, Langerød A, Green A, Provenzano E, Wishart G, Pinder S, Watson P, Markowetz F, Murphy L, Ellis I, Purushotham A, Børresen-Dale AL, Brenton JD, Tavaré S (2012). The genomic and transcriptomic architecture of 2,000 breast tumours reveals novel subgroups. Nature.

[22] Lønning PE, Knappskog S, Staalesen V, Chrisanthar R, Lillehaug JR (2007). Breast cancer prognostication and prediction in the postgenomic era. Ann Oncol.

[23] Rehman FL, Lord CJ, Ashworth A (2010). Synthetic lethal approaches to breast cancer therapy. Nat Rev Clin Oncol.

[24] Lønning PE, Knappskog S (2012). Chemosensitivity and p53; new tricks by an old dog. Breast Cancer Res.

[25] Silwal-Pandit L, Vollan HKM, Chin SF, Rueda OM, McKinney SE, Osako T, Quigley D, Kristensen VN, Aparicio S, Børresen-Dale AL, Caldas C, Langerød A (2014). Tp53 mutation spectrum in breast cancer is subtype specific and has distinct prognostic relevance. Clin Cancer Res.

[26] Lønning PE, Knappskog S (2013). Mapping genetic alterations causing chemoresistance in cancer: identifying the roads by tracking the drivers. Oncogene.

[27] Paluch-Shimon S, Friedman E, Berger R, Papa MZ, Daadiani M, Friedman N, Shabtai M, Zippel D, Gutman M, Golan T, Catane R, Yosepovich A, Mekel Mondiani T, Kaufamn B (ᅟ). Does pathologic complete response predict for outcome in *brca* mutation carriers with triple-negative breast cancer?. J Clin Oncol.

[28] Lønning PE (2004). Genes causing inherited cancer as beacons to identify the mechanisms of chemoresistance. Trends Mol Med.

[29] Baselga J, Campone M, Piccart M, Burris HA, Rugo HS, Sahmoud T, Noguchi S, Gnant M, Pritchard KI, Lebrun F, Beck JT, Ito Y, Yardley D, Deleu I, Perez A, Bachelot T, Vittori L, Xu Z, Mukhopadhyay P, Lebwohl D, Hortobagyi GN (2012). Everolimus in postmenopausal hormone-receptor-positive advanced breast cancer. New Engl J Med.

[30] Rugo HS, Hortobagyi GN, Piccart-Gebhart MJ, Burris HA, Campone M, Noguchi S, Perez AT, Deleu I, Shtivelband M, Provencher L, Masuda N, Dakhil SR, Anderson I, Chen D, Damask A, Huang A, McDonald R, Taran T, Sahmoud T, Baselga J (2013). Correlation of molecular alterations with efficacy of everolimus in hormone-receptor-positive, her2-negative advanced breast cancer: results from bolero-2. J Clin Oncol.

[31] Stites EC (2012). The response of cancers to BRAF inhibition underscores the importance of cancer systems biology. Sci Signal.

[32] Diaz LA, Bardelli A (2014). Liquid biopsies: genotyping circulating tumor DNA. J Clin Oncol.

[33] Schmitt CA, Fridman JS, Yang M, Lee S, Baranov E, Hoffman RM, Lowe SW (2002). A senescence program controlled by p53 and p16ink4a contributes to the outcome of cancer therapy. Cell.

